# Testing Sentinel-1 SAR Interferometry Data for Airport Runway Monitoring: A Geostatistical Analysis

**DOI:** 10.3390/s21175769

**Published:** 2021-08-27

**Authors:** Valerio Gagliardi, Luca Bianchini Ciampoli, Sebastiano Trevisani, Fabrizio D’Amico, Amir M. Alani, Andrea Benedetto, Fabio Tosti

**Affiliations:** 1 Department of Engineering, Roma Tre University, Via Vito Volterra 62, 00146 Rome, Italy; luca.bianchiniciampoli@uniroma3.it (L.B.C.); fabrizio.damico@uniroma3.it (F.D.); andrea.benedetto@uniroma3.it (A.B.); 2 University IUAV of Venice, Dorsoduro 2206, 30123 Venezia, Italy; strevisani@iuav.it; 3 School of Computing and Engineering, University of West London (UWL), St Mary’s Road, Ealing, London W5 5RF, UK; Amir.Alani@uwl.ac.uk (A.M.A.); Fabio.Tosti@uwl.ac.uk (F.T.)

**Keywords:** satellite remote sensing, airport runway monitoring, ESA Sentinel 1 (C-Band) SAR data, Multi-Temporal SAR Interferometry (MT-InSAR), Persistent Scatterers Interferometry (PSI), geostatistics, kriging interpolation, topographic levelling, Airport Pavement Management System (APMS)

## Abstract

Multi-Temporal Interferometric Synthetic Aperture Radar (MT-InSAR) techniques are gaining momentum in the assessment and health monitoring of infrastructure assets. Amongst others, the Persistent Scatterers Interferometry (PSI) technique has proven to be viable for the long-term evaluation of ground scatterers. However, its effectiveness as a routine tool for certain critical application areas, such as the assessment of millimetre-scale differential displacements in airport runways, is still debated. This research aims to demonstrate the viability of using medium-resolution Copernicus ESA Sentinel-1A (C-Band) SAR products and their contribution to improve current maintenance strategies in case of localised foundation settlements in airport runways. To this purpose, “Runway n.3” of the “Leonardo Da Vinci International Airport” in Fiumicino, Rome, Italy was investigated as an explanatory case study, in view of historical geotechnical settlements affecting the runway area. In this context, a geostatistical study is developed for the exploratory spatial data analysis and the interpolation of the Sentinel-1A SAR data. The geostatistical analysis provided ample information on the spatial continuity of the Sentinel 1 data in comparison with the high-resolution COSMO-SkyMed data and the ground-based topographic levelling data. Furthermore, a comparison between the PSI outcomes from the Sentinel-1A SAR data—interpolated through Ordinary Kriging—and the ground-truth topographic levelling data demonstrated the high accuracy of the Sentinel 1 data. This is proven by the high values of the correlation coefficient (*r* = 0.94), the multiple R-squared coefficient (*R*^2^ = 0.88) and the Slope value (0.96). The results of this study clearly support the effectiveness of using Sentinel-1A SAR data as a continuous and long-term routine monitoring tool for millimetre-scale displacements in airport runways, paving the way for the development of more efficient and sustainable maintenance strategies for inclusion in next generation Airport Pavement Management Systems (APMSs).

## 1. Introduction

Monitoring the structural integrity of transport infrastructures, such as highways, railways and airfields, is a priority for national authorities and asset administrators to guarantee the structural integrity, ensure the operational safety and prevent infrastructure damage and deteriorations prior to any expensive rehabilitation or structural failure [1,2].

Worldwide, dramatic events related to the vulnerability of transport infrastructures to natural hazards (e.g., earthquakes, subsidence and landslides) and endogenous events (e.g., the end of pavements’ service life, an increase in the traffic loads, the aging of materials, rebar corrosion) highlighted the importance of routine monitoring and the proper planning of maintenance activities. Countries with a transportation network system highly exposed to the effects of major natural events are actively investing money to promote more effective asset management procedures. This is the case of Italy, where the Ministry for Transport and Infrastructures has urged to implement new measures for a continuous infrastructure monitoring though the provision of dedicated guidelines [3].

Within this context, several non-destructive testing (NDT) technologies and ground-based sensors are used for infrastructure subsidence monitoring. Amongst others, accelerometers, strain gauges, wireless network systems, ground penetrating radar (GPR) and the ground-based interferometric radar systems, have proven effective for the provision of denser information and their incorporation into more functional asset management systems, as reported in Table 1.

The main limitations from a stand-alone use of these technologies can be the difference in the multi-scale source of respective datasets, the multi-temporal and the multi-spatial resolution required for the monitoring of infrastructures, a relatively limited land coverage linked with main constraints from specific working principles; the limited repeatability of measurements in time, and the high costs of monitoring at the network level [20,21].

To overcome these drawbacks, innovative integrated approaches have been developed and applied for the monitoring of civil engineering structures and infrastructures [22,23,24,25]. However, costs of the equipment remain substantially high, and the on-site operations are difficult to implement at the network level, due to budget and administrative constraints. To tackle these limitations, various innovative satellite-based remote sensing technologies, i.e., the Interferometric Synthetic-Aperture Radar (InSAR) [26,27,28,29,30] have been successfully applied in the last few years for the monitoring of transportation infrastructures. Main advantages of these techniques are related to the provision of very dense and frequently updated arrays of data as well as to the availability of historical time-series of displacements accessible through numerous available archives.

Amongst the main limitations, we can mention (i) the need to collect datasets with different orbit geometries or, alternatively, using ground-truth reference points for the actual vertical and horizontal components of the displacements, and (ii) high computational efforts required for the processing of SAR imagery at the network level, especially in case of long-period investigations and high-frequency datasets (i.e., X-Band). Regarding the latter point, the use of C-Band data stands as a more easily accessible information by the end-users for large-scale analyses and the measurements of settlements at the centimetre scale. In fact, use of the medium resolution imagery allows for lighter data processing and management flows, and it permits to investigate wider portions of the territory and transportation networks.

Nevertheless, several limitations can still be pointed out about using medium-resolution data for transport infrastructure monitoring, including (i) the ground pixel resolution, which does not allow allocating a detected PS displacement from a randomly given object to its actual position on the ground, and (ii) the accuracy of the measurements, due to the limited frequency range of the sensors. Under specific conditions, especially in urban areas with buildings located in the vicinity of transport infrastructures, assigning the PS information directly to relevant infrastructure elements can be a difficult task. Hence, use of the medium ground-resolution in this case remains a challenge, due to the limited size of the infrastructures in relation to the maximum available monitoring resolution.

Within this framework, this study reports a geostatistical analysis for the investigation of the spatial variability characteristics of C-Band data and their accuracy in comparison to conventional ground-based methods. To elaborate, medium resolution C-Band data have been compared to the information collected through topographic levelling for the monitoring of vertical subsidence in airport runways. Furthermore, the sampling geometry and the spatial variability of the C-Band data are compared with same features from X-Band products, which are referred to as the benchmark information in terms of precision and accuracy of displacements [31].

## 2. Displacement Monitoring Techniques and Data-Analysis Methods for Airport Infrastructure Management: Background and Open Issues

Conventional and novel techniques for the monitoring of displacements in transport infrastructures and airport runways are presented in this section. In detail, the topographic levelling and the LiDAR techniques will be here referred to as “conventional techniques”, as opposed to the satellite-based Multi-Temporal SAR Interferometry (MT-InSAR) methods, i.e., referred to as “advanced techniques” for network-scale monitoring.

### 2.1. Topographic Levelling

Levelling is a relatively straightforward operation in topographic surveying based on the variation measurement of the elevation between consecutive surveys. In transport infrastructure monitoring, use of the geometric levelling relies on achieving the height difference from readings on levelling staffs [32].

According to [33], the topographic levelling allows monitoring the long-term settlements of an infrastructure from the variation in the vertical position of a target across multiple surveys. This is a relative measurement referred to a single stable point or to multiple stable points, which are typically indicated as topographic benchmarks. In the last few years, several levelling-based investigations of civil infrastructures have been reported in the literature [32,33]. Amongst others, the main limitations include (i) a limited productivity due to a main constraining condition of measuring marks individually, (ii) the necessity to close the infrastructure during the inspections, (iii) the impossibility to perform surveys in adverse weather conditions, and (iv) the difficulty in the provision of a clear line of sight between consecutive targets in a survey.

### 2.2. Multi-Temporal SAR Interferometry (MT-InSAR) 

The working framework of the multi-temporal InSAR technique relies on statistical analyses of the signal emitted by the on-satellite sensor and back-scattered by a network of coherent targets on the ground, i.e., the Persistent Scatterers (PSs). 

The selection of PSs is based on the signal amplitude information associated to the pixels of a SAR image, as the original interferometric phase (Δ*ϕ*) ranges in the interval (–π ÷ π). More specifically, the phase (*ϕ*) is the key information in any interferometric application, as it relates to different phase components, as follows:

*ϕ* = *ϕ_flat_* + *ϕ_topo_* + *ϕ_def_* + *ϕ_atm_* + *ϕ_noise_* (1)

where *ϕ _flat_*, *ϕ _topo_*, *ϕ _def_*, *ϕ _atm_*, and *ϕ _noise_* are the phase components related to the reference surface, topography, deformations, atmospheric delay and noise, respectively. 

**Figure 1:**
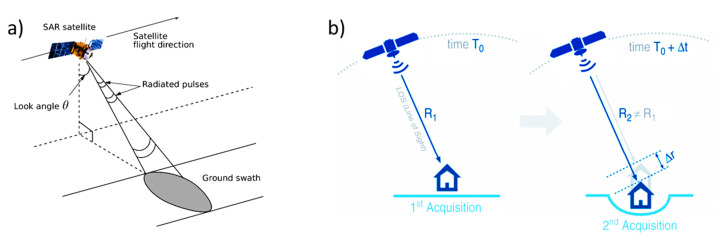
(**a**) SAR sensor and acquisition layout; (**b**) Main working principles of the PS-InSAR technique, detecting the phase-shift (Δ*r*) produced by a ground deformation.

Displacements are detected by relating the signal phase variation between progressive acquisitions to the motion of a target under investigation (Figure 1). In detail, the general approach is to compare a dataset of N SAR images, collected at different times on the same area, computing the interferometric phase (Δ*ϕ*) as the phase difference, for each N-1 pairs referred to the same master image. The component related to the ground deformations (*ϕ_def_*) must be worked out from Equation (1), which is proportional to the sensor-to-target distance difference (Δ*r*) divided by the wavelength (λ) of the SAR sensor, as expressed by Equation (2):

(2) $$\;\Delta \phi_{def}=\frac{4\pi }{\lambda }\Delta r\;$$

A correct detection of PSs strongly depends on the phase stability, which is expressed as the PS coherence. The pixel selected as a candidate PS must have an amplitude large enough to dominate the resolution cell, which remains stable in terms of its electromagnetic response (e.g., back-scattering features are stable in time) across a significant dataset of SAR images. Thus, the clutter component slightly affects the phase (*ϕ*) due to the statistical relationship between the calibrated amplitude stability and the phase stability.

This approach allows to measure target displacements across multiple satellite acquisitions by the separation between the phase shift from the ground motions and those related to atmospheric, topographic and signal noise contributions [26,27,28,29,34]. The MT-InSAR technique is computationally affordable, and it has proven effective in the assessment of displacements and the detection of critical sections [28,29]. 

In the last few years, several MT-InSAR techniques (i.e., InSAR [26,27], Small Baseline Subset (SBAS) [24,25,26,27,28], SqueeSAR [25]) relying on different approaches for the detection of PS targets (i.e., coherence, or amplitude stability index based), have been used for the monitoring of transport infrastructures, such as railways, highways, [35,36,37,38,39], bridges, tunnels [40,41,42,43,44,45,46], and airport runways [47,48].

However, SAR satellites can only detect displacements in the Line-of-Sight (LoS) of the SAR sensor, with reference to the specific orbit-related incident angle. Therefore, the detected displacement is a component of the real displacement on the ground. Different methods have been proposed in the literature to overcome this issue and to evaluate the real displacements-velocity-vector. Amongst others, this can be achieved by a combination between the LoS displacement information from two different datasets acquired in Ascending (Asc.) and Descending (Desc.) acquisition geometries [49,50]. 

#### MT-InSAR for Airport Infrastructure Monitoring: An Overview of Applications and Areas of Further Research

In the last few years, limited research has been conducted on the application of satellite interferometry to the monitoring of airport runways. Mostly, these studies did not specifically focus on their inspection, but rather on the assessment of large-scale subsidence involving airport systems, i.e., the land side and the air side. Furthermore, this research mainly aimed at relating the InSAR results to the main geological features of the areas investigated, which are usually geotechnically complex portions of territory that had been previously reclaimed to the sea. In addition to this, it is observed that a lack of research exists within the context of InSAR data management and integration in airport pavement management systems. To this effect, this section reports an overview of research about the use of InSAR techniques in airport monitoring, to highlight the main findings and identify areas of further research.

Jiang et al. [51] present a case study on the deformation monitoring and the analysis of the geological environment at the Shanghai Pudong International Airport, China. The authors used a multi-temporal InSAR approach to identify several down-lifting displacement areas. Processing was carried out on a SAR dataset composed by 15 TerraSAR-X SAR images acquired in descending geometry. A qualitative spatio-temporal analysis of the ground deformations confirmed a correlation between the InSAR measurements and the variations observed in the geological environment.

Jiang et al. [47] present an integrated analysis of SAR interferometric and geological data for the investigation of long-term reclamation settlements at the Chek Lap Kok Airport, Hong Kong. Relying on a dataset of ascending (25 scenes) and descending (22 scenes) images acquired by the ENVISAT ASAR mission (C-Band) and a sparse levelling campaign, the authors successfully related the deformation trend observed in the areas of the airport to the main geotechnical properties of the soils, with a special focus on the area reclaimed by the sea. More recently, the same settlement occurrence has been investigated by Wu et al., [52] through an extensive InSAR analysis covering two decades of observations. The authors merged information from several multi-temporal datasets collected through various space missions (i.e., ERS-2, ENVISAT ASAR, COSMO-SkyMed and Sentinel-1A). The outcomes were compared to the data collected using GNSS stations located in the airport area as well as to available geological data. The study successfully demonstrated the effectiveness of InSAR analyses in the monitoring of extensive settlements in areas reclaimed to the sea.

The same topic has been addressed by Liu et al. [53]. The authors used the SBAS InSAR approach on Sentinel-1 SAR images acquired in Ascending geometry. The scope was to monitor the ground settlements following a sea reclamation land from construction of the Xiamen New Airport, China. The study reports significant information for airport land reclamation design purposes and the next stages of the construction process.

More recently, Gao et al. [54] presented research on the monitoring of severe down-lifting deformations at the Beijing Capital International Airport, China. In this case, the authors demonstrated that settlements were not related to consolidation effects on sea reclaimed lands but mostly on the variation of the groundwater levels. An InSAR investigation into the differential settlements observed on one of the runways at the Fiumicino International Airport in Rome, Italy, has been reported by Bianchini et al. [48]. In this work, processed X-Band SAR images highlighted the presence of vertical displacements. These were compared to the outputs from a dense levelling campaign conducted over four years’ time. The limited errors confirmed that PS-InSAR analyses of high frequency images are very effective in reconstructing vertical deformations in airfield runways.

In general, existing research has widely demonstrated the effectiveness of InSAR analyses for the interpretation of geological and geotechnical features in areas of interest for airports. In addition, these studies clearly demonstrate the reliability of the InSAR technique in reconstructing the actual vertical displacements, with a very limited difference across the adopted frequency band and the acquisition geometry, given the quasi-vertical principal direction of deformations. However, gaps in knowledge and ground for further research and development are still observed concerning the satellite monitoring of airport systems and, especially, the runways. Although satellite data are in some cases compared and validated against other established technologies and methods (e.g., the use of GNSS stations or levelled points), the spatial density of this ground-truth information is very limited in most cases. This makes it difficult to assess the geostatistical significance of these data, especially at the local level and in critical sections of the airport system (e.g., in case a detailed monitoring of displacements in airport runways is required). Furthermore, the lack of PSs and ground measurement points still limits, and in some cases, it can also inhibit the provision of a continuous monitoring of runways through InSAR techniques. To elaborate, the application of conventional InSAR approaches can be constrained by limitations in the number of PSs at certain sections of runways (e.g., due to different back-scattered effects related to the roughness of the pavement) [55]. This can pave the way to new research focussing on the application of existing InSAR methods with Distributed Scatterers (DS) supported by geostatistical analyses [56,57,58].

Within this framework, it is fair to observe that the aforementioned research is mostly related to the investigation of deformation patterns at a wider scale of inspection through the application of InSAR techniques. Conversely, a more detailed assessment of the airport individual structures and infrastructures using medium resolution satellite data is still to be covered. In this regard, the provision of InSAR-related information for integration into more advanced APMSs could be crucial to predict major failures in advance and maintain airport infrastructures more effectively. 

The medium-resolution dataset analysed in this paper stems from the Sentinel-1 Mission. This is the European Imaging Radar Observatory for the Copernicus joint initiative of the European Commission (EC) and the European Space Agency (ESA). This mission is composed of a constellation of two satellites, Sentinel-1A and Sentinel-1B [59]. As a constellation of two satellites orbiting 180° apart, the mission covers the entire Earth every six days. To this extent, Sentinel-1 data are ideal for transport infrastructure monitoring purposes in view of a relatively short revisiting time and a large land coverage. On the other hand, the typical ground resolution of the C-Band imagery (i.e., 20 m of azimuth resolution and 5 m of range resolution for Sentinel-1 data) can stand as a significant limitation for applications to transport infrastructure networks and still represents an open challenge.

Within this framework, the main peculiarities and areas of novelty related to the satellite and ground-based datasets presented in this paper are summarised below:

- The comparison between measurements from satellite databases (medium and high resolutions) and ground-truth measurements from topographic levelling directly collected on an airport runway;
- Analysing a medium resolution dataset (Sentinel 1, C-Band) for the monitoring of displacements in an airport runway affected by well-known deformations, including a long-term investigation for the suitability of the Sentinel-1 sensor to detect displacements in the area of subsidence;
- The dataset peculiarities: the runway is constructed on a flat area, hence, due to the high construction standard requirements [60], this can be assumed as a horizontal structure with limited and evenly-distributed settlements. In this paper, the effectiveness of the C-Band medium resolution is tested against a scenario where settlements have formed relatively rapidly in time and are localised in certain sections.

### 2.3. Data-Analysis Methods: An Overview on Geostatistical Approaches and Areas of Further Research

Geostatistics [61,62] is a branch of statistics dealing with the analysis of spatio-temporal data representing the values of a physical-chemical property of interest. Geostatistics is characterised by a well-established theoretical background and validated approaches and it is mostly popular for the use of the interpolation Kriging-based algorithms [61]. These are widely implemented in many software packages and are fundamental tools for use in geographic information systems [62]. Beyond the interpolation algorithms, geostatistical methodologies have been also developed for exploratory spatial data analyses (ESDA) and the investigation of spatial uncertainties [61].

The kriging-based algorithms, denoted as the Best Linear Unbiased Estimators (BLUE), are designed to predict parametric values in unsampled locations. This can be achieved by using a weighted linear combination of neighbouring data, with weights designed to obtain an unbiased prediction with minimum prediction variance [63,64]. The spatial autocorrelation of data (e.g., covariance or variogram function) plays a pivotal role in the system of normal equations [63], to be resolved for calculation of the interpolation weights. To this effect, the weighting scheme is considered as an objective feature [65], as it depends on the inherent spatial—statistical structure of the data.

Geostatistics relies on the random function theory [62]. Accordingly, the spatial distribution of a physical—chemical property is considered as the outcome of a spatial random function. The bivariate spatial autocorrelation of the data is generally evaluated with a variogram (Equation (3)):

(3) 2γ(h)=Var {Z(u)−Z(u+h)}

The variogram (Equation (3)) is the semi-variance of the difference between two random variables located in two different locations, u and u+h (with **u** and **h** vectors). Under second order or, at least, intrinsic stationarity conditions [61], the semi-variance depends only on the separation **h** between pairs of locations; in these conditions, the variogram can be estimated directly by the available data $z\left(u_{\alpha }\right)$, as follows:

(4) $$\gamma \left(h\right)=\frac{1}{2N\left(h\right)}\overset{N}{\underset{\alpha =1}{\sum }}{\left[z\left(u_{\alpha }\right)-z\left(u_{\alpha }+h\right)\right]}^{2}$$

The experimental variogram (h), for a given value of h, is the half of the mean squared differences of the N(h) data pairs $z\left(u_{\alpha }\right)-z\left(u_{\alpha }+h\right)$, which are spatially separated by the vector h. Considering a set of discrete values of h (lags), it is possible to estimate a set of values of the theoretical function γ(h) from the data. The experimental variogram cannot be used directly to solve the kriging system of equations, as it should be known for every value of **h**, and its negative should be a positive definite function [61]. Accordingly, a theoretical function, selected amongst a family of authorised models, should be inferred from the experimental variogram. This can be achieved using diverse approaches, such as the ordinary/weighted least squares or the maximum likelihood approaches [66].

A standard geostatistical analysis tailored for interpolation purposes is characterised by three main stages: (1) ESDA; (2) Inference of the variogram; (3) Interpolation and accuracy assessment. A relevant part of the methodology relies on the ESDA, which includes the study of the spatial autocorrelation. The ESDA is a fundamental multi-purpose step, which is capable of highlighting connections between computed spatial statistics, domain-related knowledge and information linked with the data acquisition procedures.

The main objectives of this step of the analysis are as follows:

- The detection of local and global outliers; the detection of potential non-stationarity in spatial variability (e.g., the presence of a trend);
- An assessment of the necessity to transform the data due to highly skewed distributions (e.g., by means of log and box-cox transformations);
- The detection of spatial continuity anisotropy.

As reported above, a variogram can be estimated directly from the raw data only under, at least, intrinsic spatial stationarity conditions (i.e., stationarity of the mean and of the variogram). Consequently, during ESDA, it is crucial to detect non-stationarity in the data, which could be related to the presence of trends and/or non-stationarity in local variance. Moreover, the analysis of a variogram can provide further information on data errors and their short-range variability, as well as on the spatial support of samples [64,65,66,67].

The second step of the analysis is focused on the inference of a variogram model. In this stage, the fitting of the experimental variogram is performed according to a weighted least squares approach, using the algorithm implemented in the Gstat R-package [66]. In the fitting, the weights for a specific lag h are directly proportional to the number of sample pairs and inversely proportional to the squared distance.

The third stage of the analysis is focused on the application of the Kriging-based interpolation [61]. The selection of a specific Kriging algorithm is dependent on the spatial-statistical structure of the data, considering both the primary variable of interest as well as any potential secondary correlated variable. The potential presence of a trend is an important factor in selecting the most suitable Kriging algorithm. In this context, it is convenient to decompose the random variable Z(u) into a residual stochastic component R(u) and a deterministic component m(u) (5):

(5) Z(u)=R(u)+m(u)

R(u) is a second order (i.e., stationarity of expected value, variance and variogram) stationary random function, with an expected value equal to zero; m(u) is the trend, generally representing long-range spatial patterns. The trend/residual decomposition permits to handle the non-stationarity of Z(u) by filtering out the trend component m(u). The Ordinary Kriging (OK) and the Universal Kriging (UK) algorithms [61], including regression kriging and kriging with external drift [61,62,63,64,65], use this approach to deal with spatial trends. The OK is one of the most flexible and practical kriging algorithms, being capable to handle a trend m(**u**) changing smoothly in the domain of investigation, when it can be considered constant inside a moving local subdomain W(**u**).

Further advantages of using kriging algorithms are as follows: (i) the possibility of evaluating the prediction uncertainty, by an estimation of the prediction variance, and (ii) the capability of interpolating across non-punctual spatial supports, by means of the Block Kriging algorithm. The selection of areal (in 2D) or volumetric (in 3D) interpolation supports allows reducing the estimation variance by decreasing the spatial resolution [61,62,63,64].

Within this framework, the main peculiarities and areas of novelty related to the geostatistical analyses presented in this paper are linked with the possibility of comparing variograms from InSAR and levelling data. More specifically, the availability of ground-truth levelling data allows the following operations:

- To investigate into their spatial variability across the runway, which is affected by displacements of different scale (size and spatial distribution);
- To optimise the fitting model for interpolation purposes;
- To explore the variability characteristics of the satellite data (medium and high resolution).

## 3. Aim and Objectives

The main aim of the research presented in this paper is to investigate into the applicability of medium-resolution (C-Band) satellite remote sensing imagery (e.g., data from the Sentinel 1 A mission) for the long-term routine monitoring of millimetre-scale displacements in airport runway pavements. To this effect, a case study is presented where “Runway n.3” of the “Leonardo Da Vinci International Airport” in Fiumicino, Rome, Italy, has been monitored with medium-resolution Sentinel-1A and high-resolution COSMO SkyMed satellite data, as well as with topographic levelling. The runway has been taken as an explanatory case study in view of historical geotechnical settlements affecting its area.

To quantify the displacements obtained through the satellite-based technologies and validate them with on-site topographic levelling measurements, a geostatistical approach has been adopted based on two major investigation goals. The first is related to the sampling geometry of the Sentinel data, which is characterised by clustering and relatively low spatial density. Accordingly, with these data the accuracy of the values interpolated on the grid nodes or at points of interest (e.g., the levelling points) can be significantly affected by the interpolation approach adopted. A second important aspect is the necessity to study the spatial continuity structure of the Sentinel data, also in comparison with the ground-based levelling data and the COSMO-SkyMed data. 

To achieve the main aim of this research, set objectives are as follows:

- To measure and evaluate the suitability of Sentinel 1 (C-Band) SAR data for monitoring airport runway pavements displacements on a multi-year temporal scale through the satellite-based PSI monitoring technique;
- To compare the results obtained by the PSI technique to the measurements collected via a dense topographic levelling campaign, through a geostatistical analysis;
- To evaluate the feasibility of the proposed geostatistical analysis as a reliable investigation approach for the comparison of satellite-based and ground-based displacement information, as well as a tool for the integration of satellite-based information within next generation APMS to improve upon current maintenance strategies.

## 4. Methodology

The methodology presented in this paper is based on two major stages. The acquisition and processing of satellite and ground-based data for displacement monitoring in airport runways are first presented, followed by a geostatistical study. 

**Figure 2:**
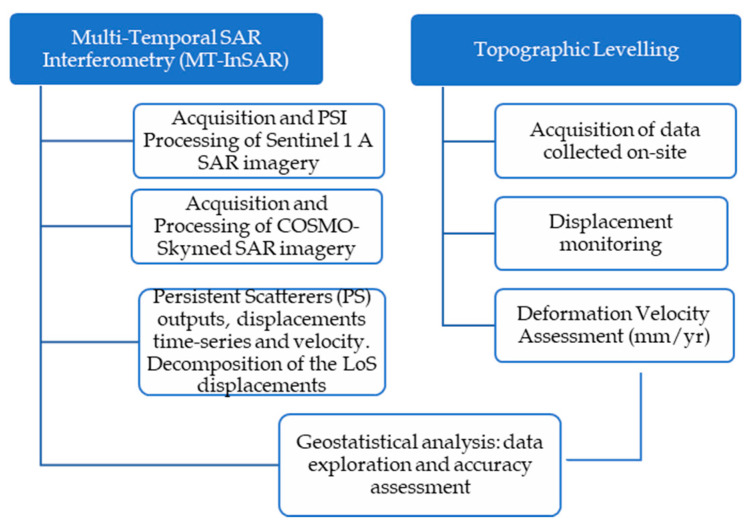
Flow-chart of the implemented methodology: the MT-InSAR, the topographic levelling and the geo-statistical investigative approach.

To elaborate, a first stage of the methodology is aimed at evaluating the average velocity values of the displacements (mm/yr) for the detected Persistent Scatterers (PSs). This procedure was conducted using both Sentinel 1-A and high-resolution COSMO-SkyMed SAR datasets. More specifically, the decomposition of displacements from Sentinel 1 data in the vertical and horizontal directions is evaluated. Parallel to this, the acquisition and analysis of displacements collected on-site by topographic levelling are implemented. For the second stage of the presented methodology, a geostatistical study is reported for the exploratory spatial data analysis and the interpolation of the Sentinel-1 SAR data The implemented methodology is showed in the flow-chart in Figure 2.

### 4.1. Implemented Displacement Monitoring Techniques and Data Exploration Approach

#### 4.1.1. Topographic Levelling: Data Acquisition Methods

The first stage of a topographic levelling survey consists in the acquisition of the data, which is performed by collecting data directly on site. To this effect, the runway must be closed to service for the entire duration of the survey. The relative position of the identified topographic nets is measured with reference to a fixed stable ground control point, taken outside the investigated runway and not affected by displacements over time. Measurements are carried out at regular time intervals, e.g., monthly. As a result, the displacements observed at any survey can be monitored, and the deformation velocities can be calculated with a sub-millimetre accuracy. A major advantage of this well-established method is in the high accuracy and reliability of the information collected. The main disadvantage is related to the high costs of the data collection stage on-site and its complex logistics. Traffic on runways must be in fact interrupted during the surveys (i.e., the runway must be closed), hence, it cannot be carried out at regular short intervals in time.

In this study, a five-year dataset of topographic levelling measurements was collected with a time-frequency of one year, for a total number of 466 levelled points. For each levelled point of the dataset, it was possible to assess the trend of displacements and, therefore, to compute the average deformation velocity in the reference period.

#### 4.1.2. PSI Data Processing

Two SAR imagery datasets with different operating frequencies were acquired. Details of these datasets are given in Section 5.2. Then, the imageries were processed by the application of the PSI technique.

The PSI technique performed in this paper operates by the application of a multi-stage approach, which was based on a sequence of the following chronological steps [26,28,29]:

- Creation of the “Connection Graphs” where a Master image is selected to allow the identification of the connections for the formation of the interferograms. Then, a statistical analysis of the amplitudes of the electromagnetic response is performed on a pixel-by-pixel basis to compute the Amplitude Dispersion Index. The reference master image was selected amongst those acquired in the middle of the temporal and perpendicular baseline domain, to minimise space and temporal decorrelations. Therefore, the corresponding interferograms for each pair of master-slave images are computed.
- The second step is based on the identification of the Persistent Scatterer Candidates (PSCs), selected by computing the amplitude dispersion index values relative to each pixel. The PSCs are pixels, associated to the resolution cell of the SAR sensor, with a value of stability index exceeding a fixed threshold (typically of 0.25). The interferometric phase Δ*ϕ_i_* is computed for any PSC, at any *i*th interferogram.
- The atmospheric phase contributions (*ϕ*_atm_) as well as, the orbital and noise-related effects (*ϕ*_noise_) are evaluated and removed from the interferometric phase (Δ*ϕ_i_*), to identify the phase-shift uniquely related to the range variations. To elaborate, the topographic phase (*ϕ*_topo_) is removed using the Digital Elevation Model (DEM) acquired in the framework of the Shuttle Radar Topographic Mission (SRTM), with a pixel resolution of 3 arc seconds (90 × 90 m). This is made available by the National Aeronautics and Space Administration (NASA) in partnership with the United States Geological Survey (USGS). The DEM resolution is adopted considering that the area investigated for the airport runway is flat, and the phase values are slightly affected by this parameter.

The above-listed processing phases have been applied by means of the “Interferometric Stacking Module” of the Software SARscape [68], which is integrated in the Software ENVI and licensed within the context of the “STRAIN2” EOhops project (ID 53071), funded by the European Space Agency (ESA).

As a result of the above steps, stable reflectors (i.e., the PSs) can be detected over the inspected area. At the end of the analysis, the PSs with a temporal coherence above 0.7 were eventually selected. For each PS, the LoS velocity, the displacement time-series and heights have been estimated using a linear trend model of the deformations.

### 4.2. Geostatistical Analysis

Geostatistics has been used to evaluate the spatial statistical structure and the accuracy of Sentinel 1 SAR data in monitoring runway displacements (see Section 2.3). Concerning the ESDA process, statistical summaries and spatial statistical tools, such as the classed post maps [62], the variogram cloud [69,70], the directional variograms and the variogram maps [62] have been computed in this study. The analysis has been carried out using the Gstat library [65], implemented for the R statistical programming environment [71], and relevant exploratory tools of the “Geostatistical analyst” package of the GIS software Arcmap 10.7 (Esri). The geostatistical analysis implemented in this study is developed according to the following main steps:

- ESDA: analysis of the spatial sampling geometry, statistical summaries, spatial explorative analysis and analysis of the spatial continuity by means of experimental variograms, with identification of the main directions of the anisotropy.
- Inference of a variogram model: fitting of the experimental variogram with a variogram model, considering the main directions of the anisotropy.
- Interpolation and accuracy assessment: data interpolation using the fitted variogram model and the appropriate kriging algorithm, considering the spatial statistical structure of the data.

In this research, a punctual OK was selected as the interpolation algorithm at the levelling points, due to the spatial statistical characteristics of the dataset. The quality of the interpolation parameters and the consistency of the inferred variogram model were evaluated by means of the leave-one-out Cross-Validation (CV) diagnostics [62], including the root-mean-square error (RMSE), the root mean square standardised error (RMSSE), and the statistical and spatial distribution of the errors. The interpolation of Sentinel 1 SAR data at the levelling points allows evaluating the accuracy of the Sentinel 1 SAR data in the investigated domain. Moreover, a comparative analysis of the spatial continuity of Sentinel 1, COSMO-SkyMed, and levelling data can provide further information on the accuracy of the Sentinel 1 data with respect to the COSMO-SkyMed data. 

As a main novelty of the proposed geostatistical approach, it is worth mentioning the optimisation of the model, due to a large availability of ground-truth levelling points. This allows defining a maximum distance of interpolation and investigating into the directional variograms. Hence, the accuracy of the geostatistical model used for data analysis and interpretation purposes can be improved.

## 5. Case Study

### 5.1. Area of Investigation

**Figure 3:**
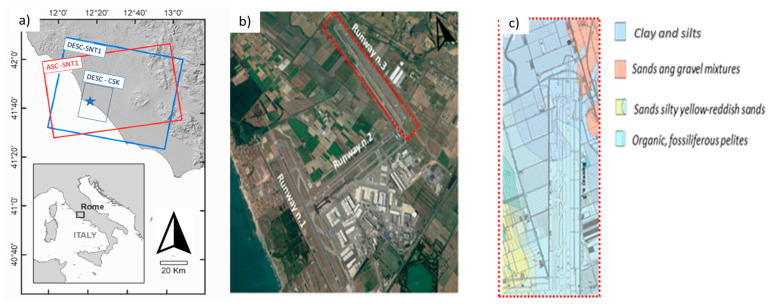
(**a**) Area of interest and footprint of the selected Sentinel-1 master scenes for both ascending and descending tracks. ALOS World DSM used as background; (**b**) Satellite view of the “Leonardo Da Vinci” International Airport and location area of Runway n.3 (Google Earth Image, 2015); (**c**) Geological setting of Runway n.3.

The study area is located at the “Leonardo Da Vinci” International Airport in Fiumicino, Rome, Italy. The airport carries most of the intercontinental air traffic to and from the Italian territory, and it is ranked as the main national airport and one of the most trafficked at the international level. The airport airside system is composed of three runways with a capacity of 90 aircraft movs/hour, including take-off and landing operations. However, its original layout was formed by only two runways. Following an increasing demand in the air traffic volumes, a third runway (i.e., Runway n.3 in Figure 3) was in fact realised in the ‘70s and located in the North-East area of the airport, alongside the North–South direction. Furthermore, reports in the “Airport Development Plan” predict an increase in the air traffic stream from approximately 40 million passengers/year (year 2014) to 65 million passengers/year in 2027, bringing the current airside system capacity to 120 aircraft movs/h. Additionally, the “Master Plan 2030” [72] set new directions for the construction of a fourth runway, the eastern expansion of the aircraft aprons, the construction of new flight infrastructures and a new terminal system to support an expected increase in the air traffic demand.

Within this framework, it is worth mentioning that maintenance of the existing asset and plans for its future expansion are heavily affected by a well-known and active subsidence of the foundation soils. This is due to the presence of clayey and silty geological layers with poor load-bearing capacity, as shown in the geological maps of the area reported in Figure 3c. [73]. In more detail, the first 50 m of the subsoil are characterised by a high sedimentary heterogeneity with a prevalence of a palustrine sedimentary environment (cohesive and organic soils) in the south-east runway sector and a fluvial sedimentary environment (with presence of clay and silt) in the north-western runway sector [73]. This lithological heterogeneity has caused severe differential settlements on Runway n. 3.

### 5.2. Displacement Monitoring Techniques: Equipment and Datasets

#### 5.2.1. Satellite SAR Datasets

The SAR images were acquired in the framework of the Sentinel 1A mission, in the ascending (i.e., with the satellite orbiting South to North, looking in the East direction) and descending (i.e., with the satellite orbiting North to South, looking in the West direction) acquisition geometries, whereas the used COSMO-SkyMed SAR products rely on a single acquisition geometry only (descending). The first step consists in identifying the displacements detected in the LoS of the satellite sensors. The datasets were processed using the PSI technique, as described in Section 4.1.2. The Copernicus Sentinel-1 SAR data covered the period between April 2017 and December 2019. The X-Band SAR products, covering the period between November 2016 and December 2019, were acquired by the COSMO-SkyMed mission. The main characteristics of the Sentinel-1 and COSMO-SkyMed missions are reported in the (Table 2).

The COSMO-SkyMed system operates in X-Band at a frequency of 9.6 GHz (wavelength of 3.1 cm). This allows to achieve a ground-resolution square cell of 3 m size and—under ideal conditions—a millimetre accuracy for the measurements. 

#### 5.2.2. Topographic Levelling Equipment

A standard geometric levelling survey of the runway was conducted using the DNA03 Digital Level system, manufactured by Leica. The elevation measurements were collected by means of a 2 m high levelling invar rod. The main features of the employed levelling system are summarised in Table 3.

**Figure 4:**
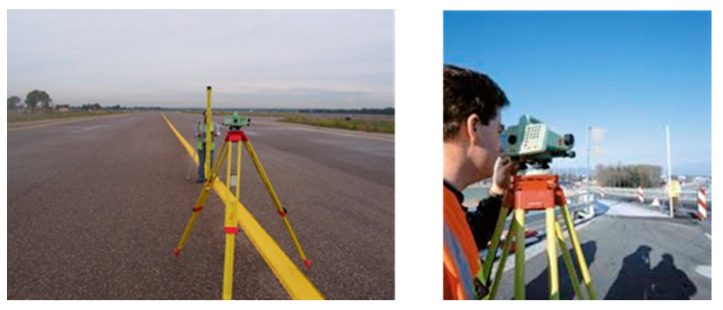
Example of a topographic levelling survey for airport runway monitoring [74].

The collected nets were automatically compensated, with an average Squared Root Mean Error (SQRM) of 0.94 mm. The starting and ending point of the measurements was a levelling benchmark situated in the North-West corner of the runway area, which was verified to have a stable elevation. This point was connected to the high precision levelling net developed by the Istituto Geografico Militare (IGM), through a levelling line with an average accuracy of 1.0 mm/km. Tests were performed every year from 2015 to 2019 and covered five sections along the runway with a transversal spacing of 15 m and a longitudinal length equal to the entire runway development. As shown in Figure 4, the levelled points were spaced 15 m on the transverse direction, summing up to a total of five measurements for every inspected cross-section of the runway. In turn, cross-sections were spaced 60 m across the longitudinal direction (i.e., the take-off/landing direction). This spacing was further decreased up to 10 m at critical sections, i.e., areas subject to heavy displacements. 

## 6. Results and Discussion

### 6.1. Displacement Monitoring Techniques: Persistent Scatterers Interferometry (PSI) and Topographic Levelling Investigations

**Figure 5:**
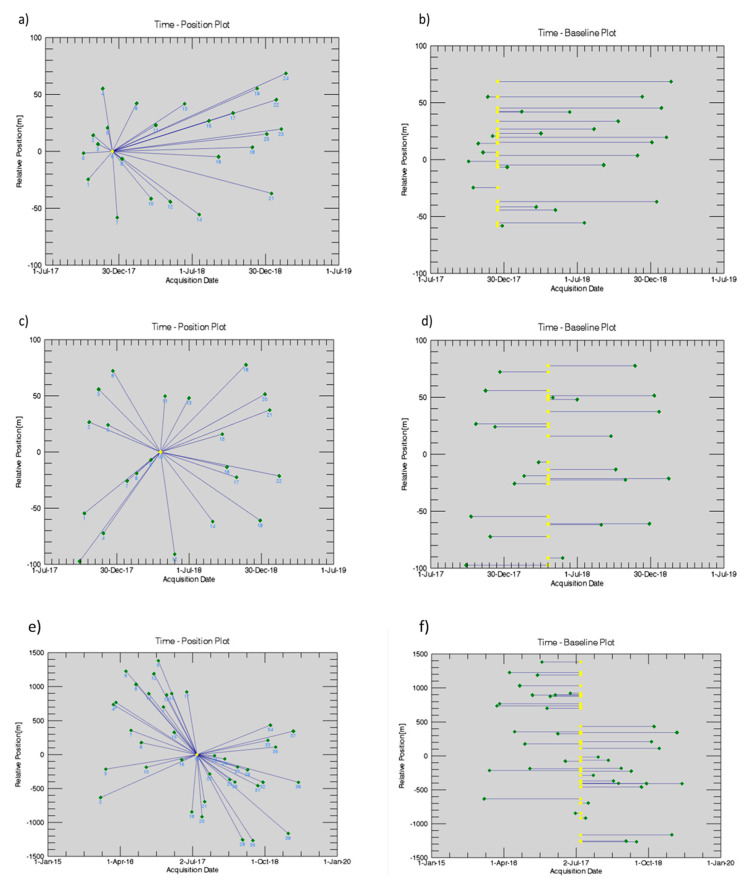
Connection graphs of: Time-Position (**a**,**c**,**e**) and Time-Baseline (**b**,**d**,**f**) plots of: Sentinel 1A Ascending (**a**,**b**); Sentinel 1A Descending (**c**,**d**); COSMO−SkyMed Descending (**e**,**f**) datasets. Data display is in relation to the master image (indicated in yellow) and the slaves (green dots), which were identified to calculate the interferograms.

The connection graphs containing detailed information on the SAR acquisitions, including the time of acquisition and the baseline, are reported in Figure 5. More specifically, the Time-Baseline and the Time-Position plots are reported, where the yellow dots represent the Master image selected in the stack of each database, and the green dots are the slaves selected to compute the differential interferograms.

**Figure 6:**
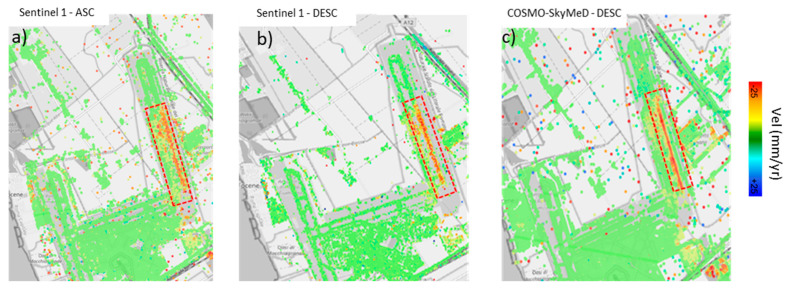
PSI results in the Study Area identified in the LoS: ascending (**a**) and descending (**b**) geometry for the Sentinel-1; descending geometry for the COSMO−SkyMed (**c**) datasets. Data display is performed in relation to the average trend of velocity. Ground subsidence is clearly visible in the area of interest (marked with a dashed rectangular red area).

The outcomes of the PSI processing phases are reported in Figure 6, in terms of the PS outputs from the C-Band and the X-Band SAR datasets. 

The PS outputs were imported into a GIS environment and displayed as a function of the annual average velocity of motion. The green points are referred to the stable scatterers with a displacement velocity ranging from −4 mm/yr to +4 mm/yr. Figure 6 provides an overview of the average LoS annual velocities estimated from the Sentinel and the COSMO-SkyMed data.

The figure colourmap refers to displacements in the range [−25 ÷ +25] mm/year. Across the entire airport area, it was possible to detect several PSs showing a coherent trend of deformation for both the SAR datasets. A number of PSs, mostly located in proximity to Runway n.3, are shown to be affected by a down-lifting trend of deformations. In detail, Figure 6 clearly shows that Runway n. 3 can be divided in two distinct areas from North to South direction, i.e., a stable area in the northern part of the runway (displacements reported in green), and a southern area affected by higher settlements (displacements reported in red).

It is worth noting that, as the SAR sensor can detect displacements in reference to the incident angle of the LoS only, the detected displacement is a component of the real velocity vector on the ground.

**Figure 7:**
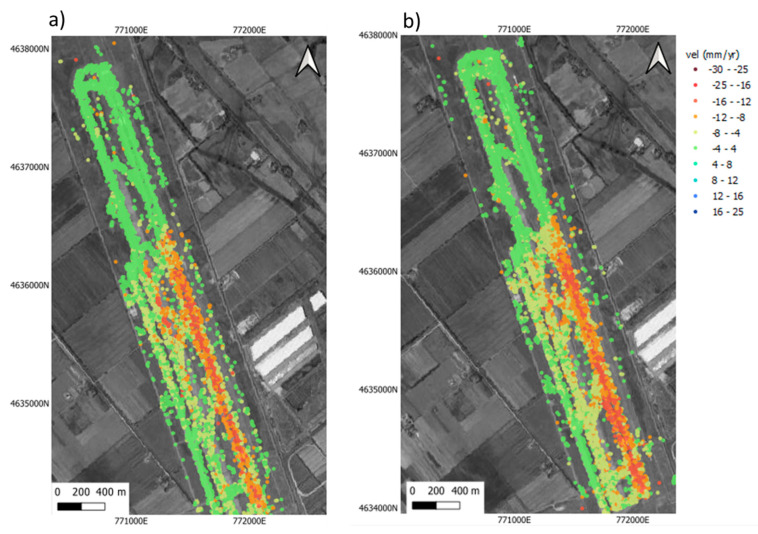
PSI results identified in the study area in the LoS in (**a**) ascending and (**b**) descending geometries of the Sentinel 1 dataset. The down−lifting area affected by subsidence, represented by red dots, is clearly visible in both the datasets. The basemap is taken from Google maps.

To investigate the decomposition and the real direction of the displacements, the PSs obtained by the MT-InSAR processing of the Sentinel 1 data were post-processed and compared (Figure 7). These data were acquired in both the acquisition geometries.

Regardless of the specific acquisition geometry, the LoS displacement (*D_los_*) is a projection of a three-dimensional displacement vector [47,49,50]:

(6) $$D_{los}=d_{v}\cos\left(\vartheta \right)-d_{e}\cos\left(\beta \right)\sin\left(\vartheta \right)-d_{n}\sin\left(\beta \right)\sin\left(\vartheta \right)$$

with *d_v_*, *d_e_* and *d_n_* being the vertical, eastern and northern components of the displacement, respectively, *ϑ* and *β* being the incident angles and the azimuth of the radar, respectively. Given the conditions of (i) SAR acquisitions not being affected by PS displacements in the N-S direction and (ii) satellite orbits almost parallel to the N-S direction, it is reasonable to assume this component as negligible. This reduces the number of unknowns in Equation (6). 

Under this assumption, it is possible to evaluate the actual vertical component of the PS displacements if both the acquisition geometries are available, by solving the following system of equations [47,49]:

(7) $$\left[\begin{array}{c} d_{ASC}\\ d_{DESC} \end{array}\right]=\left[\begin{array}{cc} \cos\left(\vartheta_{ASC}\right) & -\sin\left(\vartheta_{ASC}\right)\cos\left(\beta_{ASC}\right)\\ \cos\left(\vartheta_{DESC}\right) & -\sin\left(\vartheta_{DESC}\right)\cos\left(\beta_{DESC}\right) \end{array}\right]\left[\begin{array}{c} d_{v}\\ d_{e} \end{array}\right]$$

where *ASC* and *DESC* refer to the ascending and descending acquisition geometries, respectively, and $d_{v}$ and $d_{e}$ are the vertical and the horizontal components of the displacements, respectively. 

As in most of the cases it is no possible to associate a randomly given PS obtained from an acquisition geometry to another PS with the same coordinates but different acquisition geometry, geostatistical approaches, e.g., the use of interpolated grids, are required for matching the datasets. Nevertheless, the use of interpolated information, especially in case of medium-resolution Sentinel 1 datasets, implies that new uncertainties could be introduced and affect the interpretation of results. This can stand as a limit of the PSI technique for pavement inspections, given the relatively high spatial variability of distresses and faults. Accordingly, prior to the application of Equation (7) to the investigated PS database, a comparative analysis of the Sentinel-1 data acquired with different orbit geometries was carried out. Hence, this allows assessing the impact of neglecting the horizontal displacement component from the analysis of displacements. This assumption agrees with the flat conditions for the surveyed area and the correlation between the loading mode and type of expected displacements on the runway. Specifically, for $d_{e}$ *=* 0 Equation (7) can be solved as *d_ASC_ = d_DESC_*, i.e., it is possible to evaluate the model suitability in neglecting $d_{e}$ by comparing displacements in the LoS from both the acquisition geometries.

**Figure 8:**
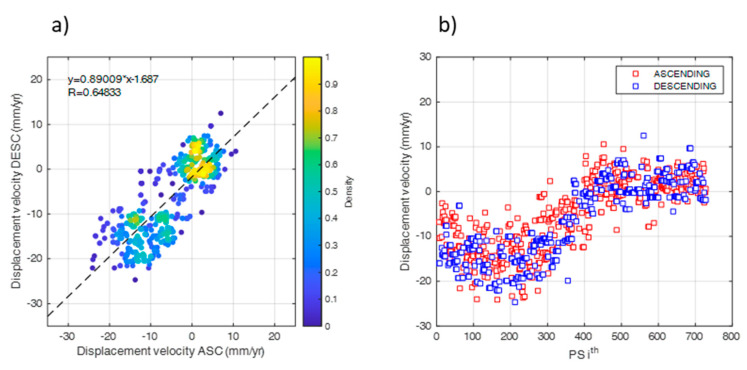
(**a**) Scatter−plot and (**b**) regular plot of the comparison between displacement velocity values of the PSs acquired in the two acquisition geometries.

To that intent, each PS_ASC_ was compared in terms of displacement velocity (mm/yr) to the mean value of the PS_DESC_ located in a radius of 20 m from the PS_ASC_ position, which is in line with the ground resolution of the sensor. The result of this operation is shown in Figure 8. 

The slope of the linear regression in Figure 8a was found relatively close to the bisector line, showing an acceptable value of the correlation coefficient (*R* = 0.62) and distribution of the errors (*μ =* 0; *σ =* 4.8 mm/yr). This is also related to the effect of the averaging operations carried out on the PS_ASC_. The analysis of the results showed that the vertical component of the displacement is dominant with respect to the horizontal component. Hence, this was neglected from the statistical analyses carried out in this paper. Accordingly, the vertical displacement has been calculated for each PS as:

(8) $$d_{v}=\frac{D_{DESC}}{\cos\left(\theta_{DESC}\right)}$$

**Figure 9:**
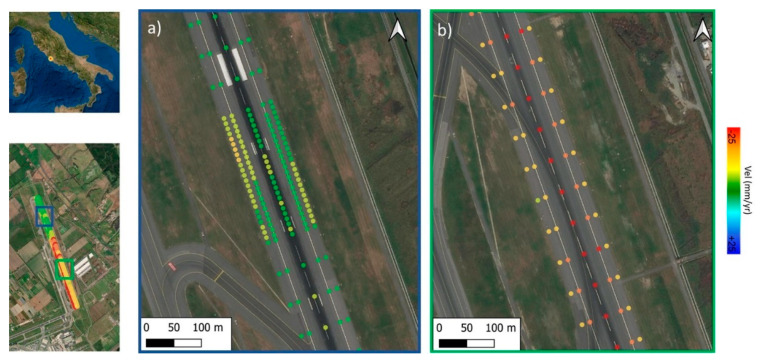
Layout of the levelling data collected on site along Runway n.3: (**a**) Density of the measuring points on the ground in the area interested by subsidence (North region of the runway); (**b**) Density of the measuring points in the stable area of the runway (South region of the runway).

Therefore, ground-truth displacements by topographic levelling on Runway n.3 (see Section 5.2.2) were used to compute the accuracy of the geostatistical model derived by the InSAR processing on the C-Band dataset. The output of these analyses allowed detecting and quantifying millimetre-scale displacements and their average deformation velocity in the time range investigated (Figure 9). This information was crucial for a validation of the displacements detected by the PSI technique.

### 6.2. The Geostatistical Analysis

**Figure 10:**
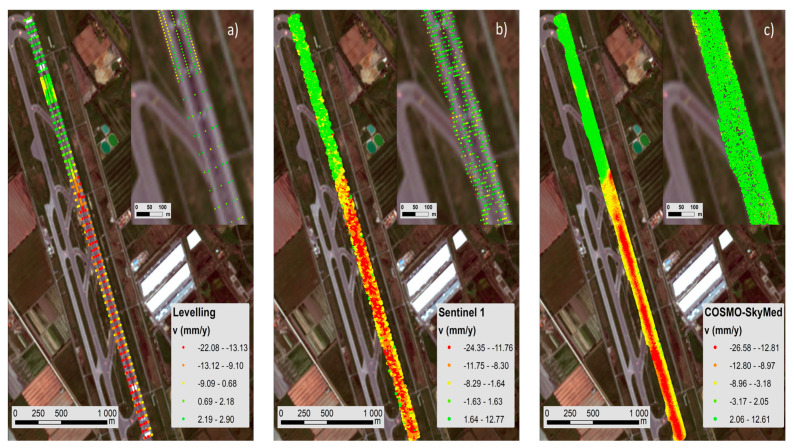
Classed post maps (quantile classification) of the ground displacements recorded by (**a**) Levelling; (**b**) Sentinel-1; (**c**) COSMO-SkyMed. The basemap is a satellite orthophoto from the Sentinel 2 sensor (ESA-Copernicus).

A buffer of 30 m was used for the geostatistical analysis of the data on the runway. The chromatic quantile classification of the mean velocity values (mm/yr) in Figure 10, distributing the set of values into groups with same number of values, represents the spatial density of the information obtained by (a) Levelling, (b) Sentinel-1 and (c) COSMO-SkyMed methods.

The PSs derived by the PSI processing of the Sentinel-1 and the COSMO-SkyMed SAR datasets are characterised by different sampling networks, both in terms of spatial sampling density and spatial clustering. This is related to the diverse operating frequencies providing different pixel resolutions. In the case of the Sentinel-1 imagery, the data are clustered along lines oriented in the NE-SW direction; the mean spacing values are 14 m and 3.9 m in the NW-SE and NE-SW directions, respectively.

**Figure 11:**
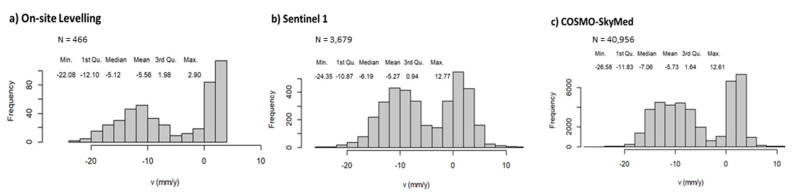
Histograms and main statistics of Levelling (**a**), Sentinel 1 (**b**), and COSMO-SkyMed (**c**) data.

Similar to the Levelling and the COSMO-SkyMed data, the Sentinel 1 data are also characterised by a clear bimodal distribution, as shown in Figure 11, due to different settlement trends between the North and South areas of the runway. From a spatial statistical viewpoint, it was considered as not necessary to divide the dataset in two further subsets, i.e., the South zone (high subsidence) and the North zone (low subsidence), as reported in Figure 10. To elaborate, the interactive analysis of the variogram cloud and the directional variograms developed for the two zones did not highlight any remarkable difference between the north and the south areas. Moreover, it is worthy of mention that a stratified approach [61] would not contribute significantly to improve the quality of the results, whereas it could increase the complexity of the analysis, due to the large amount of data available.

Nevertheless, it is fair to say that in other circumstances, e.g., studies involving larger areas with a higher heterogeneity in the spatial patterns of the ground deformations, stratified or locally adaptive approaches [75] could be instead required, including a major complexity of the adopted approach and software implementation.

**Figure 12:**
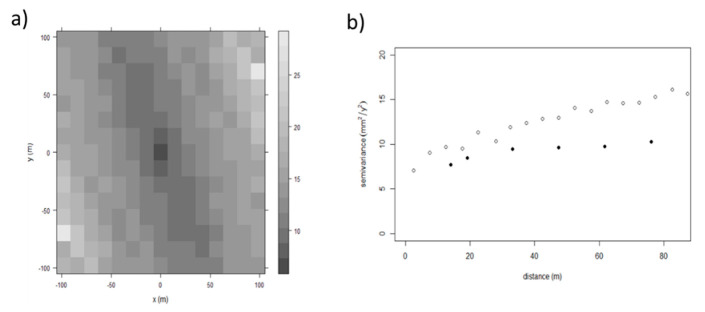
(**a**) Variogram map and (**b**) directional variograms (black dots direction 340°, white 70°) of the Sentinel 1 data, showing a higher spatial continuity in the direction of the runway (i.e., 340°).

**Figure 13:**
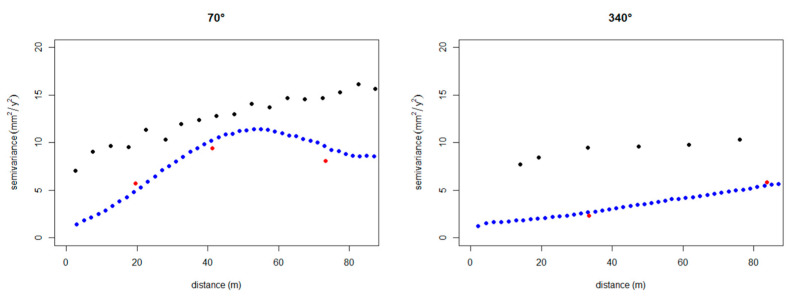
Comparison between the directional variograms (empty circle 70° and full circle direction 340°), of the Sentinel 1 (black), the COSMO-SkyMed (blue) and the topographic levelling (red) data.

Sentinel-1 data (Figure 12) are characterised by an anisotropy in their spatial continuity, with the direction of the maximum spatial continuity aligned with the runway direction (i.e., direction 340°) and the minimum spatial continuity in the transversal direction (i.e., direction 70°). This observation has been confirmed also for the Levelling and the COSMO-SkyMed data. The possibility to attribute this anisotropy to the presence of a trend has been also evaluated. However, the spatial patterns of settlements have proven rather complex to be modelled by the use of simple polynomial functions [61]. Accordingly, this anisotropy was considered as related to the stochastic component of the random function. The validity of this assumption can be considered as acceptable, since the calculation of the variogram was conducted for relatively short distances (up to 80 m), to reduce the impact of potential long-range trends and considering the limited width of the runway (Figure 13).

The analysis of the directional variograms for the Sentinel 1 data allowed their comparison with the semi-variance obtained by topographic levelling. This operation is possible due to the availability of a very dense dataset of ground-truth levelled points throughout the full cross-sectional development of the airport runway. Following an iterative investigative procedure, a geostatistical analysis has been performed for the Sentinel 1 data, the COSMO-SkyMed and the ground-truth levelling data to define a maximum value for the interpolation distance.

The high nugget and variability values of the Sentinel-1 data inevitably implies that the kriging interpolations are characterised by relatively high prediction variances, with a minimum prediction variance of 7 mm^2^/y^2^ (i.e., a standard deviation of 2.65 mm/y).

**Figure 14:**
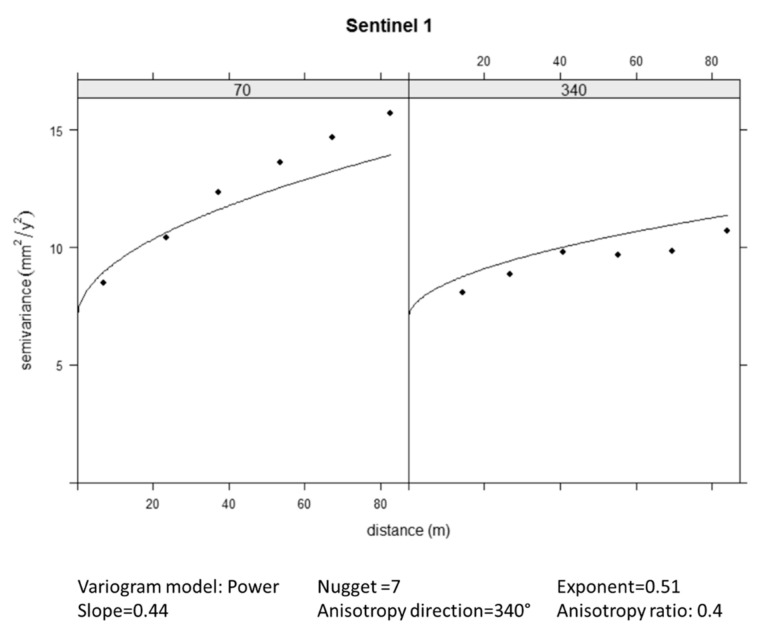
Fitting of the experimental variogram with a Power model.

The experimental variogram has been fitted by an anisotropic Power model, which is an unbounded variogram (Figure 14). Given the spatial characteristics of the data, an OK algorithm has been selected to interpolate the Sentinel-1 data at the Levelling section points. 

To investigate the capability of the Sentinel-1 data in detecting the actual ground displacements, a geostatistical analysis is conducted considering the potential impact of the interpolation uncertainty. In this stage, a denser database of ground-truth levelling data is a fundamental condition for an effective calibration of the geostatistical parameters (e.g., the maximum distance of interpolation, the anisotropy, etc.) as well as for the proper selection of the fitting power model. 

**Figure 15:**
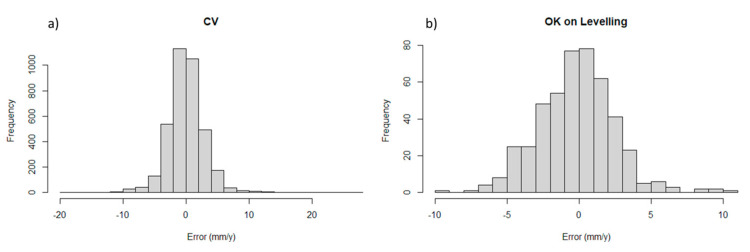
(**a**) Histogram of the Cross-Validation (CV) error; (**b**) histogram of the error computed in terms of the velocity difference (*V*_Sentinel_ —*V*_Levelling_).

The interpolation has been conducted with a search neighbourhood of 60 m using a minimum of 15 data (with less data the prediction is not performed). The reference parameters have been defined after different trials, considering cross-validations results and the spatial-statistical structure of the data. In particular, the search neighborhood was kept small to reduce the potential impact of long-range trends, especially in the transverse direction (70°) to the runway. The consistency of the interpolation parameters has been evaluated by means of a cross-validation (Figure 15). 

This reports relatively good diagnostics (Table 4), including the absence of a spatial correlation in the residuals for the modelled distances. For distances longer than 80 m in the direction 70°, signs of a spatial structure in the residuals are identified, due to the unmodelled long-range trends. 

**Figure 16:**
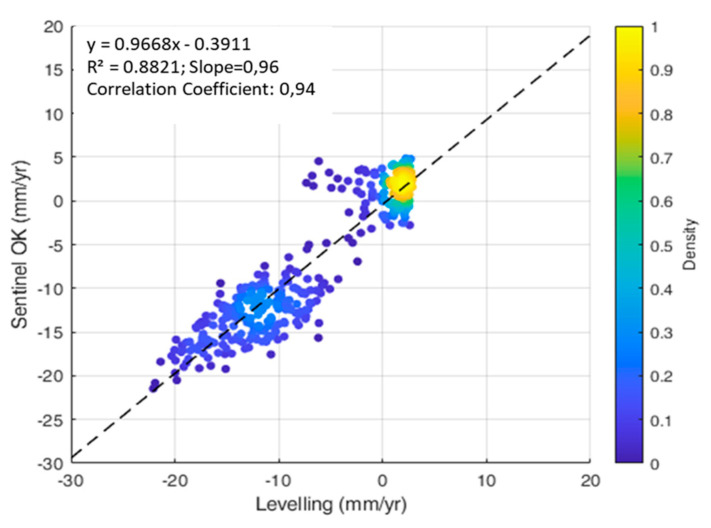
Scatter plot of the interpolated Sentinel-1 deformations versus the levelling deformations, with regression line and related fitting diagnostics.

Furthermore, a comparison between the PSI outcomes from the Sentinel-1A SAR data—interpolated through an Ordinary Kriging—and the ground-truth topographic levelling data demonstrated a high accuracy of the Sentinel 1 data. This is proven by the high values of the correlation coefficient (*r* = 0.94), the multiple *R*-squared coefficient (*R^2^* = 0.88) and the Slope value (0.96), as reported in Figure 16.

The Sentinel data have been interpolated at the levelling section points, allowing evaluating the difference between settlements (i.e., *V*_Sentinel_ − *V*_Levelling_) measured by the two approaches. It is worth to emphasise that, due to a relatively spatial dense sampling geometry of the Sentinel data, the prediction standard deviation has a limited variation (from 2.9 to 3.3 mm/y), which is most likely due to the nugget. 

The evaluation of the accuracy of the Sentinel dataset compared to the COSMO-SkyMed is also depicted by the directional variograms in the two main directions with the ones calculated from levelling and COSMO-SkyMed data. Levelling and COSMO-SkyMed data, for the same distances, have coherent values of the variogram. 

Moreover, the COSMO-SkyMed data are characterized by a very low nugget of approximately 1 mm^2^/y^2^. Assuming the nugget to be completely due to the data uncertainty, this means a standard deviation of 1 mm/y. Differently, the experimental variogram values of the Sentinel-1 data (represented by the black dots in Figure 13) are shifted with respect to the values from other datasets. The nugget is also much higher with an approximate value of 7 mm^2^/y^2^, implying a lower accuracy of the data. 

The variogram characteristics of the Sentinel-1 data are unexpected to some extent. The larger support of the Sentinel-1 footprint should in fact lead to a variogram regularisation [75] and, hence, to lower values compared to the levelling or the COSMO-SkyMed data. In turn, these should be representative of a much smaller spatial support. The general higher spatial variability of the Sentinel-1 data indicates the presence of higher noise, which can be most likely related to accuracy constraints in the deformation measurements, (associated to the C-Band frequency of the SAR sensor), or the location of the PSs in the resolution cell. 

**Figure 17:**
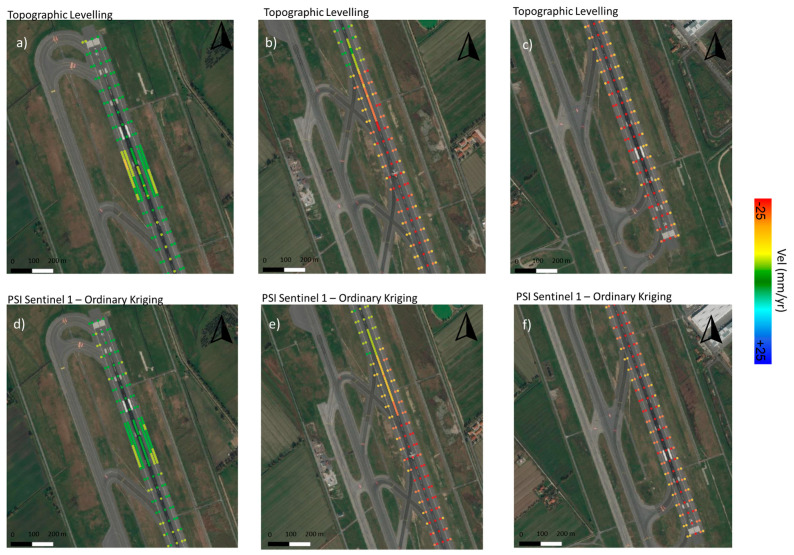
Comparison between the velocity obtained by topographic levelling (top) and the vertical components of PS-InSAR derived by Sentinel 1 SAR data, interpolated by an Ordinary Kriging (bottom), relative to (**a**–**d**) low (from 2600 m to 3200 m in N direction), (**b**–**e**) intermediate (from 1900 m to 2600 m in N direction), and (**c**–**f**) severe (from 850 m to 1620 m in N direction) displacement conditions.

As a qualitative assessment of the reliability of the analysis, Figure 17 shows a comparative view of the displacement velocity values (mm/yr) given by both topographic levelling and the vertical component of the Sentinel-1 PSI, interpolated by an Ordinary Kriging model. Therefore, it was possible to compute the velocity values, at the exact positions measured by topographic levelling. A visual comparison of the results, grouped into low (Figure 17a–d), intermediate (Figure 17b–e), and severe (Figure 17c–f) displacement conditions, preliminary indicates a remarkable reliability of the satellite data.

**Figure 18:**
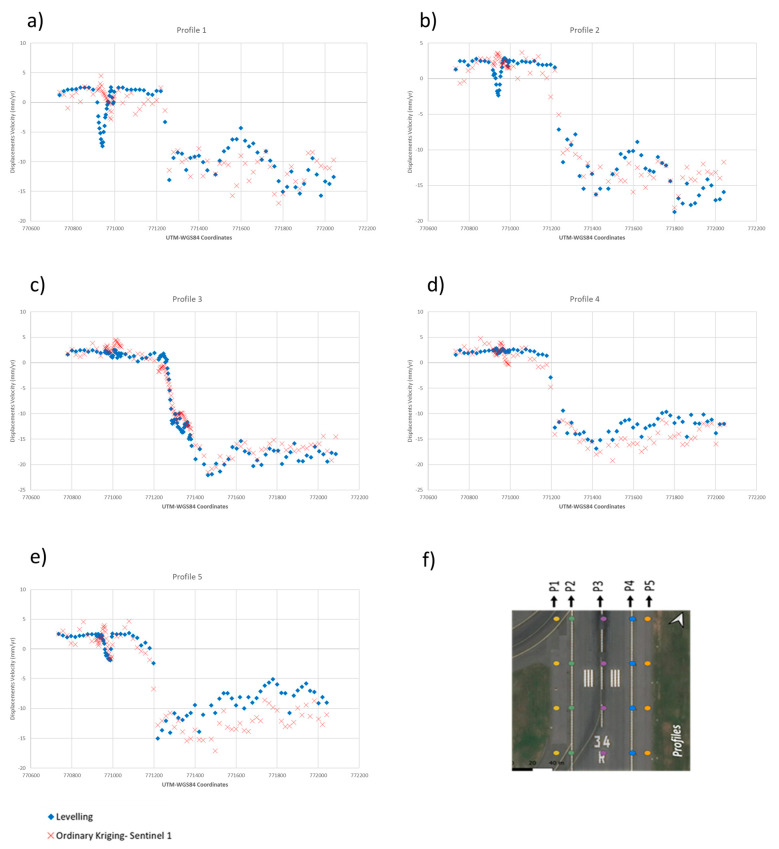
Displacement velocity trends of the vertical components of the Sentinel-1 PSI data interpolated by an Ordinary Kriging (red cross markers) and longitudinal survey profiles (**a**–**e**) P1 to P5 by topographic levelling (blue squared markers); (**f**) representation of the profile along the runways.

As a quantitative comparison of the above observations, Figure 18 shows the displacement velocity of the survey profiles for the vertical component of the Sentinel-1 PSI data, interpolated by Ordinary Kriging and the longitudinal survey profiles by topographic levelling against the space (WGS84 N Coordinate). It is possible to note that as the displacement trend reaches values higher than 15 mm/year (e.g., see Figure 18b,c), the PSI method turns out to slightly underestimate the deformation rate. However, besides this specific on-site topographic levelling observation, Figure 18 widely confirms the effectiveness of the Sentinel-1 data processed by the PSI technique and interpolated by an Ordinary Kriging method, in reconstructing the actual deformation pattern on the inspected airport runway.

## 7. Conclusions and Future Developments

This study investigates into the reliability of the C-Band Sentinel-1 mission data and the capability of the Persistent Scatterers Interferometry (PSI) remote-sensing technique to be used as an enhanced methodology for the monitoring of airport runways. 

To this effect, a PSI analysis was developed to monitor the surface deformations of “Runway n.3” at the “Leonardo Da Vinci International Airport” in Fiumicino, Rome, Italy. With reference to the methodology discussed in this paper, the presented Multi-Temporal Interferometric Synthetic Aperture Radar (MT-InSAR) technique was effective at detecting areas subject to potential active subsidence and differential down-lifting displacements.

The main conclusions from this research can be summarised as follows:

- The Sentinel1 (C-Band) SAR datasets, processed by means of the PSI technique, allow detecting airport runway displacements and quantify their velocity of motion (mm/yr) with high accuracy and correlation levels (e.g., correlation coefficient (*r* = 0.94)), compared to established on-site topographic levelling data.
- The proposed geostatistical analysis based on the Ordinary Kriging (OK) approach can be successfully implemented to compare results achieved by the application of the PSI technique to medium-resolution Sentinel-1 data with the measurements collected using the ground-based topographic levelling method. This is proven by the high values of the multiple *R*-squared coefficient (*R*^2^ = 0.88) and a Slope of 0.96.
- The presented geostatistical analysis has proven effective in comparing satellite-based and ground-based displacement information for airport runway monitoring. The relatively dense information gathered through the InSAR technique as well as the controlled conditions and the strict compliance to high standards of pavement quality and the operations in airport traffic management lends itself to be incorporated in specialist geostatistical investigations of millimetre-scale structural displacements. The information can be crucial for inclusion in next generation Airport Pavement Management Systems (APMSs).

Future research could task itself with additional geostatistical analyses for the structural monitoring of airport runways using integrated non-destructive ground-based technology for surface (e.g., laser scanner [9]) and subsurface (e.g., Ground Penetrating Radar (GPR), Falling Weight Deflectometer (FWD) [14,15,16,76]) characterisation of displacements in runways and the improvement of their safety operating conditions. 

## Figures and Tables

**Table 1:** Non-destructive testing (NDT) technologies and sensors for subsidence and displacement monitoring.

NDT Technology	References
Accelerometers	[4,5,6]
Strain Gauges	[7]
Wireless Network Systems	[8]
Laser Scanner	[9,10]
Global Position System (GPS)	[11]
Ground Penetrating Radar (GPR)	[12,13,14,15,16,17]
Levelling data	[18]
Ground-based Interferometer	[19]

**Table 2:** Main features of the SAR imagery dataset from the Sentinel 1 ESA (European Space Agency) and the COSMO-SkyMed missions—Italian Space Agency (ASI).

Satellite Missions	Sentinel 1A	COSMO-SkyMed
Band	C-Band	X-Band
Property	European Space Agency (ESA)	Italian Space Agency (ASI)
Reference Time Period	April 2017–December 2019	November 2016–December 2019
Acquisition Geometry	Descending/Ascending	Descending
Frequency/Wavelenght	5.4 GHz/λ = 5.5 cm	9.6 GHz/λ = 3.1 cm
Range Resolution	5 m	3 m
Azimuth Resolution	20 m	3 m
Acquisition mode	Interferometric Wide Swath (IW)	Stripmap HIMAGE
Processing Level	L1—Single Look Complex	L1A- Single look Complex Slant
Number of Images	25 Desc./23 Asc.	39 Desc.
Sub-Swath	IW3 for Desc. Geometry/ IW2 for Asc. Geometry	-
Mean Incidence Angle (rad/degrees)	Desc.: 0.75/42.97 Asc.: 0.67/38.39	Desc: 0.46/26.5 -

**Table 3:** Main features of the topographic levelling equipment.

Topographic Levelling Equipment	Leica DNA 03
Measuring Time	Operator dependent
Measuring Range	Up to 110 m
Levelling Accuracy	0.3 mm/km
Compensator	Pendulum with magnetic damping

**Table 4:** Results of the CV of the PSI derived by Sentinel 1 interpolated by Ordinary kriging, and the Error between OK Sentinel 1 and topographic levelling.

**Statistical Parameters of the Power Model of the Ordinary Kriging Sentinel 1—Cross Validation (CV)**						
RMSE (mm/yr): 3.04	RMSSE: 0.99					
	Min	1st Quartile	Median	Mean	3rd Quartile	Max
Error (mm/yr)	−19.94	−1.63	−0.07	−0.01	1.57	26.81
Absolute Error (mm/yr)	0	0.73	1.59	2.13	2.86	26.81
**Statistical Parameters: Ordinary Kriging Sentinel Error on Topographic Levelling (mm/yr)**						
RMSE (mm/yr): 2.67						
	Min	1st Quartile	Median	Mean	3rd Quartile	Max
Error (mm/yr)	−9.47	−1.84	−0.14	−0.21	1.32	10.69
Absolute Error (mm/yr)	0.01	0.73	1.58	2.03	2.82	10.69

## References

[1] Chang P.C., Flatau A., Liu S.C. (2003). Review Paper: Health Monitoring of Civil Infrastructure. Struct. Health Monit..

[2] Nourzad S.H.H., Pradhan A. (2016). Vulnerability of Infrastructure Systems: Macroscopic Analysis of Critical Disruptions on Road Networks. J. Infrastruct. Syst..

[3] Italian Ministry of Infrastructure and Transport (2020). Guideilines for the Classification and Management of the Risk, the Evaluation of the Safety and the Monitoring of the Existing Bridges. www.mit.gov.it/sites/default/files/media/notizia/2020-05/1_Testo_Linee_Guida_ponti.pdf.

[4] Cavalagli N., Kita A., Falco S., Trillo F., Costantini M., Ubertini F. (2019). Satellite radar interferometry and in-situ measurements for static monitoring of historical monuments: The case of Gubbio, Italy. Remote. Sens. Environ..

[5] Meng X., Dodson A.H., Roberts G.W. (2007). Detecting bridge dynamics with GPS and triaxial accelerometers. Eng. Struct..

[6] Chen K., Lu M., Fan X., Wei M., Wu J. Road condition monitoring using on-board Three-axis Accelerometer and GPS Sensor. Proceedings of the 2011 6th International ICST Conference on Communications and Networking in China (CHINACOM).

[7] Olund J., DeWolf J. (2007). Passive Structural Health Monitoring of Connecticut’s Bridge Infrastructure. J. Infrastruct. Syst..

[8] Chae M.J., Yoo H.S., Kim J.Y., Cho M.Y. (2012). Development of a wireless sensor network system for suspension bridge health monitoring. Autom. Constr..

[9] Barbarella M., Di Benedetto A., Fiani M., Guida D., Lugli A. (2018). Use of DEMs Derived from TLS and HRSI Data for Landslide Feature Recognition. ISPRS Int. J. Geo-Inf..

[10] Riveiro B., Morer P., Arias P., de Arteaga I. (2011). Terrestrial laser scanning and limit analysis of masonry arch bridges. Constr. Build. Mater..

[11] Sato H.P., Abe K., Ootaki O. (2003). GPS-measured land subsidence in Ojiya City, Niigata Prefecture, Japan. Eng. Geol..

[12] Alani A.M., Aboutalebi M., Kilic G. (2013). Applications of ground penetrating radar (GPR) in bridge deck monitoring and assessment. J. Appl. Geophys..

[13] Alani A.M., Aboutalebi M., Kilic G. (2014). Integrated health assessment strategy using NDT for reinforced concrete bridges. NDT E Int..

[14] Benedetto A., Tosti F., Ciampoli L.B., D’Amico F. (2017). An overview of ground-penetrating radar signal processing techniques for road inspections. Signal Process..

[15] Ciampoli L.B., Artagan S.S., Tosti F., Gagliardi V., Alani A., Benedetto A. A comparative investigation of the effects of concrete sleepers on the GPR signal for the assessment of railway ballast. Proceedings of the 17th International Conference on Ground Penetrating Radar (GPR).

[16] Solla M., Pérez-Gracia V., Fontul S. (2021). A Review of GPR Application on Transport Infrastructures: Troubleshooting and Best Practices. Remote Sens..

[17] Solla M., Lorenzo H., Rial F., Novo A. (2012). Ground-penetrating radar for the structural evaluation of masonry bridges: Results and interpretational tools. Constr. Build. Mater..

[18] Mossop A., Segall P. (1997). Subsidence at The Geysers Geothermal Field, N. California from a comparison of GPS and leveling surveys. Geophys. Res. Lett..

[19] Stabile T.A., Perrone A., Gallipoli M., Ditommaso R., Ponzo F.C. (2013). Dynamic Survey of the Musmeci Bridge by Joint Application of Ground-Based Microwave Radar Interferometry and Ambient Noise Standard Spectral Ratio Techniques. IEEE Geosci. Remote Sens. Lett..

[20] Tosti F., Alani A.M., Benedetto A., Loizos A., Soldovieri F. (2020). Guest Editorial: Recent Advances in Non-destructive Testing Methods. Surv. Geophys..

[21] Tosti F., Benedetto A., Ciampoli L.B., D’Amico F., Plati C., Loizos A. (2020). Guest Editorial: Data Fusion, integration and advances of non-destructive testing methods in civil and environmental engineering. NDT E Int..

[22] Solla M., Lagüela S., Fernández N., Garrido I. (2019). Assessing Rebar Corrosion through the Combination of Nondestructive GPR and IRT Methodologies. Remote Sens..

[23] Lagüela S., Solla M., Puente I., Prego F.J. (2018). Joint use of GPR, IRT and TLS techniques for the integral damage detection in paving. Constr. Build. Mater..

[24] Alani A.M., Tosti F., Bianchini Ciampoli L., Gagliardi V., Benedetto A. (2020). An integrated investigative approach in health monitoring of masonry arch bridges using GPR and InSAR technologies. NDT E Int..

[25] Bianchini Ciampoli L., Gagliardi V., Clementini C., Latini D., Del Frate F., Benedetto A. (2020). Transport Infrastructure Monitoring by InSAR and GPR Data Fusion. Surv. Geophys..

[26] Colesanti C., Ferretti A., Prati C., Rocca F. (2003). Monitoring landslides and tectonic motions with the Permanent Scatterers Technique. Eng. Geol..

[27] Ferretti A., Fumagalli A., Novali F., Prati C., Rocca F., Rucci A. (2011). A new algorithm for processing interferometric data-stacks: SqueeSARTM. IEEE Trans. Geosci. Remote Sens..

[28] Ferretti A., Prati C., Rocca F. (2000). Nonlinear subsidence rate estimation using permanent scatterers in differential SAR interferometry. IEEE Trans. Geosci. Remote Sens..

[29] Ferretti A., Prati C., Rocca F. (2001). Permanent scatterers in SAR interferometry. IEEE Trans. Geosci. Remote Sens..

[30] Lanari R., Mora O., Manunta M., Mallorqui J.J., Berardino P., Sansosti E. (2004). A small-baseline approach for investigating deformations on full-resolution differential SAR interferograms. IEEE Trans. Geosci. Remote Sens..

[31] Bianchini Ciampoli L., Gagliardi V., Ferrante C., Calvi A., D’Amico F., Tosti F. (2020). Displacement Monitoring in Airport Runways by Persistent Scatterers SAR Interferometry. Remote Sens..

[32] Elhassan I.M., Ali A. (2011). Comparative study of accuracy in distance measurement using: Optical and digital levels. J. King Saud Univ. Eng. Sci..

[33] Cosser E., Roberts G.W., Meng X., Dodson A.H. Measuring the Dynamic Deformation of Bridges Using a Total Station. Proceedings of the 11th FIG Symposium on Deformation Measurements.

[34] Berardino P., Fornaro G., Lanari R., Sansosti E. (2002). A new algorithm for surface deformation monitoring based on small baseline differential SAR interferograms. IEEE Trans. Geosci. Remote Sens..

[35] Tosti F., Gagliardi V., D’Amico F., Alani A.M. (2020). Transport infrastructure monitoring by data fusion of GPR and SAR imagery information. Transp. Res. Procedia.

[36] Chang L., Dollevoet R.P.B.J., Hanssen R. (2017). Nationwide Railway Monitoring Using Satellite SAR Interferometry. IEEE J. Sel. Top. Appl. Earth Obs. Remote Sens..

[37] Yang Z., Schmid F., Roberts C. Assessment of Railway Performance by Monitoring Land Subsidence. Proceedings of the 6th IET Conference on Railway Condition Monitoring (RCM 2014).

[38] D’Amico F., Gagliardi V., Bianchini Ciampoli L., Tosti F. (2020). Integration of InSAR and GPR techniques for monitoring transition areas in railway bridges. NDT E Int..

[39] Ciampoli L.B., Gagliardi V., Calvi A., D’Amico F., Tosti F. (2019). Automatic network level bridge monitoring by integration of InSAR and GIS catalogues. Multimodal Sens. Technol. Appl..

[40] Gagliardi V., Benedetto A., Ciampoli L.B., D’Amico F., Alani A.M., Tosti F. (2020). Health monitoring approach for transport infrastructure and bridges by satellite remote sensing Persistent Scatterers Interferometry (PSI). Earth Resources and Environmental Remote Sensing/GIS Applications XI.

[41] Milillo P., Giardina G., DeJong M.J., Perissin D., Milillo G. (2018). Multi-Temporal InSAR Structural Damage Assessment: The London Crossrail Case Study. Remote Sens..

[42] Gagliardi V., Ciampoli L.B., D’Amico F., Alani A.M., Tosti F., Battagliere M.L., Benedetto A. (2020). Bridge monitoring and assessment by high-resolution satellite remote sensing technologies. SPIE Future Sensing Technologies.

[43] Barla G., Tamburini A., Del Conte S., Giannico C. (2016). InSAR monitoring of tunnel induced ground movements. Géoméch. Tunnelbau.

[44] Koudogbo F., Urdiroz A., Robles J.G., Chapron G., Lebon G., Fluteaux V., Priol G. Radar interferometry as an inno-vative solution for monitoring the construction of the Grand Paris Express metro network—First results. Proceedings of the World Tunnel Conference.

[45] Gagliardi V., Ciampoli L.B., D’Amico F., Alani A.M., Tosti F., Benedetto A. (2021). Multi-Temporal SAR Interferometry for Structural Assessment of Bridges: The Rochester Bridge Case Study. Airfield and Highway Pavements 2021.

[46] Gao M., Gong H., Chen B., Zhou C., Chen W., Liang Y., Shi M., Si Y. (2016). InSAR time-series investigation of long-term ground displacement at Beijing Capital International Airport, China. Tectonophysics.

[47] Jiang L., Lin H. (2010). Integrated analysis of SAR interferometric and geological data for investigating long-term reclamation settlement of Chek Lap Kok Airport, Hong Kong. Eng. Geol..

[48] Gagliardi V., Bianchini Ciampoli L., D’Amico F., Alani A.M., Tosti F., Battagliere M.L., Benedetto A. Novel perspectives in the monitoring of transport infrastructures by Sentinel-1 and COSMO-SkyMed Multi-Temporal SAR Interferometry. Proceedings of the 2021 International Geoscience and Remote Sensing Symposium, IGARSS.

[49] Fuhrmann T., Garthwaite M.C. (2019). Resolving Three-Dimensional Surface Motion with InSAR: Constraints from Multi-Geometry Data Fusion. Remote Sens..

[50] Gagliardi V., Ciampoli L.B., D’Amico F., Tosti F., Alani A.M., Benedetto A. A novel geo-statistical approach for transport infrastructure network monitoring by Persistent Scatterer Interferometry (PSI). Proceedings of the 2020 IEEE Radar Conference (RadarConf20).

[51] Jiang Y., Liao M., Wang H., Zhang L., Balz T. (2016). Deformation Monitoring and Analysis of the Geological Environment of Pudong International Airport with Persistent Scatterer SAR Interferometry. Remote Sens..

[52] Wu S., Yang Z., Ding X., Zhang B., Zhang L., Lu Z. (2020). Two decades of settlement of Hong Kong International Airport measured with multi-temporal InSAR. Remote Sens. Environ..

[53] Liu X., Zhao C., Zhang Q., Yang C., Zhang J. (2019). Characterizing and Monitoring Ground Settlement of Marine Reclamation Land of Xiamen New Airport, China with Sentinel-1 SAR Datasets. Remote Sens..

[54] Gao M., Gong H., Li X., Chen B., Zhou C., Shi M., Guo L., Chen Z., Ni Z., Duan G. (2019). Land Subsidence and Ground Fissures in Beijing Capital International Airport (BCIA): Evidence from Quasi-PS InSAR Analysis. Remote Sens..

[55] Karimzadeh S., Matsuoka M. (2020). Remote Sensing X-Band SAR Data for Land Subsidence and Pavement Monitoring. Sensors.

[56] Even M., Schulz K. (2018). InSAR Deformation Analysis with Distributed Scatterers: A Review Complemented by New Advances. Remote Sens..

[57] Shamshiri R., Nahavandchi H., Motagh M., Hooper A. (2018). Efficient Ground Surface Displacement Monitoring Using Sentinel-1 Data: Integrating Distributed Scatterers (DS) Identified Using Two-Sample t-Test with Persistent Scatterers (PS). Remote Sens..

[58] Béjar-Pizarro M., Guardiola-Albert C., García-Cárdenas R.P., Herrera G., Barra A., Molina A.L., Tessitore S., Staller A., Ortega-Becerril J.A., García-García R.P. (2016). Interpolation of GPS and Geological Data Using InSAR Deformation Maps: Method and Application to Land Subsidence in the Alto Guadalentín Aquifer (SE Spain). Remote Sens..

[59] European Space Agency (ESA). https://sentinel.esa.int.

[60] Italian Civil Aviation Authority (ENAC) (2014). Regulations for the Construction and the Exercise of the Airports.

[61] Goovaerts P. (1997). Geostatistics for Natural Resources Evaluation.

[62] Isaaks E.H., Srivastava R.M. (1989). An Introduction to Applied Geostatistics.

[63] Journel A.G. (1989). Fundamentals of Geostatistics in Five Lessons.

[64] Manson S.M., Burrough P.A., McDonnell R.A. (1999). Principles of Geographical Information Systems: Spatial Information Systems and Geostatistics. Econ. Geogr..

[65] Herzfeld U.C. (1996). Inverse theory in the earth sciences—An introductory overview with emphasis on gandin’s method of optimum interpolation. Math. Geol..

[66] Pebesma E.J. (2004). Multivariable geostatistics in S: The gstat package. Comput. Geosci..

[67] Atkinson P.M., Tate N.J. (2000). Spatial Scale Problems and Geostatistical Solutions: A Review. Prof. Geogr..

[68] (2012). SARscape Technical Description. http://www.sarmap.ch/pdf/SARscapeTechnical.pdf.

[69] Hengl T., Heuvelink G.B.M., Stein A. (2004). A generic framework for spatial prediction of soil variables based on regression-kriging. Geoderma.

[70] Ploner A. (1999). The use of the variogram cloud in geostatistical modelling. Environmetrics.

[71] R Core Team (2017). R: A Language and Environment for Statistical Computing.

[72] Italian Civil Aviation Authority (ENAC). https://www.enac.gov.it/aeroporti/infrastrutture-aeroportuali/master-plan.

[73] Manassero M., Dominijanni A. (2010). Riqualifica Strutturale di un Sistema di Piste Aeroportuali Rivista Italiana di Geotecnica. https://associazionegeotecnica.it/wp-content/uploads/2014/01/rig_310_046.pdf.

[74] Global Registration Services. www.grs.it.

[75] Walter C., McBratney A.B., Douaoui A., Minasny B. (2001). Spatial prediction of topsoil salinity in the Chelif Valley, Algeria, using local ordinary kriging with local variograms versus whole-area variogram. Soil Res..

[76] Benedetto A., D’Amico F., Tosti F. (2014). Improving safety of runway overrun through the correct numerical evaluation of rutting in Cleared and Graded Areas. Saf. Sci..

